# Comparing the immune response to a novel intranasal nanoparticle PLGA vaccine and a commercial BPI3V vaccine in dairy calves

**DOI:** 10.1186/s12917-015-0481-y

**Published:** 2015-08-21

**Authors:** Fawad Mansoor, Bernadette Earley, Joseph P. Cassidy, Bryan Markey, Simon Doherty, Michael D. Welsh

**Affiliations:** Agri-Food & Biosciences Institute, Veterinary Sciences Division, Stoney Road, Stormont, Belfast, BT4 3SD UK; Animal and Bioscience Research Department, Animal & Grassland Research and Innovation Centre, Teagasc, Grange, Dunsany, Co. Meath, Ireland; Veterinary Sciences Centre, School of Agriculture, Food Science and Veterinary Medicine, University College Dublin, Belfield, Ireland; Present address: SiSaf Ltd, Innovation Centre, Northern Ireland Science Park, Queen’s Island, Belfast, BT3 9DT UK

**Keywords:** Nanoparticle, PLGA, Livestock, Vaccine, Bovine respiratory disease

## Abstract

**Background:**

There is a need to improve vaccination against respiratory pathogens in calves by stimulation of local immunity at the site of pathogen entry at an early stage in life. Ideally such a vaccine preparation would not be inhibited by the maternally derived antibodies. Additionally, localized immune response at the site of infection is also crucial to control infection at the site of entry of virus. The present study investigated the response to an intranasal bovine parainfluenza 3 virus (BPI3V) antigen preparation encapsulated in PLGA (poly dl-lactic-co-glycolide) nanoparticles in the presence of pre-existing anti-BPI3V antibodies in young calves and comparing it to a commercially available BPI3V respiratory vaccine.

**Results:**

There was a significant (*P* < 0.05) increase in BPI3V-specific IgA in the nasal mucus of the BPI3V nanoparticle vaccine group alone. Following administration of the nanoparticle vaccine an early immune response was induced that continued to grow until the end of study and was not observed in the other treatment groups. Virus specific serum IgG response to both the nanoparticle vaccine and commercial live attenuated vaccine showed a significant (*P* < 0.05) rise over the period of study. However, the cell mediated immune response observed didn’t show any significant rise in any of the treatment groups.

**Conclusion:**

Calves administered the intranasal nanoparticle vaccine induced significantly greater mucosal IgA responses, compared to the other treatment groups. This suggests an enhanced, sustained mucosal-based immunological response to the BPI3V nanoparticle vaccine in the face of pre-existing antibodies to BPI3V, which are encouraging and potentially useful characteristics of a candidate vaccine. However, ability of nanoparticle vaccine in eliciting cell mediated immune response needs further investigation. More sustained local mucosal immunity induced by nanoparticle vaccine has obvious potential if it translates into enhanced protective immunity in the face of virus outbreak.

## Background

BPI3V is commonly involved as a pathogen related to bovine respiratory disease (BRD) in cattle and preventing bovine parainfluenza 3 virus (BPI3V) infection in calves is challenging [1]. An ideal vaccine for the prevention of BRD should limit viral infection to the upper respiratory tract (URT), and prevent its spread to the lungs [2]. However, significant obstacles to effective vaccine design in neonates is observed due to the fact that young calves typically have circulating maternally-derived antibodies and their immune system is relatively immature. Passively derived maternal antibodies can interfere with and inhibit the immune response to a vaccine in calves until their decline by 6 months of age [3]. In early neonatal life the calf’s immune system is skewed towards a Th2 type immune response as a consequence of high levels of progesterone and Th2 cytokines produced at the maternal-foetal interface [4–6]. One way of reducing the effects of maternally derived antibody on vaccine response is to deliver the vaccine directly onto the nasal mucosa [7], a route which favour the use of nanoparticles (NPs) [8].

To date, no research has been published examining the immune response of cattle to PLGA-NPs encapsulating BPI3V antigens. However, there are studies investigating immune response to PLGA-NPs in rodents [8]. The aim of the present study was to investigate and compare the humoral and cellular immune responses of artificially reared dairy calves to intranasal immunisation with BPI3V vaccines, Poly (dl-lactic co glycolide) nanoparticles encapsulating BPI3V nanoparticles (PLGA-BPI3V-NPs), and a commercially available temperature-sensitive modified live BPI3V vaccine. The dairy calves used in this study were sero-positive to BPI3V at the beginning of the study. Our hypothesis was to test if PLGA-BPI3V-NPs could induce or enhance BPI3V specific antibodies in calves with pre-existing antibodies and how this immune response would compare with that induced by a commercially available intranasal vaccine.

## Results

### Nanoparticle size, zeta potential and protein loading efficiency

Nanoparticles encapsulating BPI3V proteins calves with pre-existing antibodies and were spherical in shape and uniformly distributed. Particles were on average 225.4 nm in diameter and held a zeta potential of −22.7 mV (Table 1) with negative polarity. The encapsulation/loading efficiency of BPI3V proteins was 52.6 %, 2.1 mg of 4 mg used for encapsulation.

**Table 1:** Details of the size and zeta potential of NPs encapsulating BPI3V

PEAKS		
Diameter (nm)	Vol %	Width
225.4	100	190.3
ZETA POTENTIAL		
Mobility (u/S/V/cm)	−1.7800	
Zeta Potential (mv)	−22.7800	
Charge (fC)	−0.03650	
Conductivity (uS/cm)	11	

### Clinical assessment of calves

No clinical signs of disease were reported in calves in any of the treatment groups. Moreover, no adverse reaction to commercial or NP preparation was observed during the study Pre-existing levels of antibodies in the calves and antibody levels are shown in Tables 2 and 3.

**Table 2:** Effect of four different intranasal treatments on the nasal mucus IgA immune response (OD values ± s.d) in Friesian calves

Treatments	Pre I	1 week PI	2 weeks PI	3 weeks PI	4 weeks PI	1 week PB	2 weeks PB	3 weeks PB	4 weeks PB	Half euthanised	5 days PE	2 weeks PE	3 weeks PE	T × S
	Day 0	Day 7	Day 14	Day 21	Day 28	Day 42	Day 49	Day 56	Day 63	Day 71	Day 76	Day 85	Day 92	
Commercial vaccine	1.28^a^ ± 0.24	1.71^a^ ± 0.79	1.43^a^ ± 0.78	1.72^a^ ± 0.84	1.38^a^ ± 0.54	1.49^a^ ± 0.51	2.33^b^ ± 1.13	1.62^a^ ± 0.71	2.03^b^ ± 0.48	1.7^a^ ± 0.67	1.49^a^ ± 0.52	1.55^a^ ± 0.89	1.71^a^ ± 1	NS
Empty NPs	1.32^a^ ± 0.31	1.59^a^ ± 0.64	1.45^a^ ± 0.45	1.64^a^ ± 0.7	1.22^a^ ± 0.57	1.50^a^ ± 0.57	1.70^a^ ± 0.9	1.32^a^ ± 0.54	1.40^a^ ± 0.77	1.71^a^ ± 0.77	1.21^a^ ± 0.86	0.98^b^ ± 0.61	1.18^ab^ ± 0.78	NS
BPI3V	1.09^a^ ± 0.21	1.96^b^ ± 0.58	1.91^b^ ± 0.72	2.34^b^ ± 0.62	1.92^b^ ± 0.62	2.24^b^ ± 0.61	2.24^b^ ± 0.92	2.21^b^ ± 0.44	2.22^b^ ± 0.76	2.18^b^ ± 0.6	1.36^ab^ ± 0.4	1.49^b^ ± 0.93	1.18^a^ ± 0.87	NS
PLGA-BPI3V-NPs	1.10^a^ ± 0.3	1.73^b^ ± 0.4	1.34^ab^ ± 0.3	1.68^b^ ± 0.44	1.77^b^ ± 0.44	1.85^b^ ± 0.5	2.96^c^ ± 0.66	2.63^c^ ± 0.44	2.94^c^ ± 0.71	3.10^c^ ± 1.28	3.10^c^ ± 0.73	3.15^c^ ± 0.72	3.15^c^ ± 0.71	**

*Pre I* Pre immunisation sampling, *PI* Post immunisation, *PB* Post booster, *PE* Post euthanasia, *T × S* treatment × sampling time interaction. Levels of significance: NS = *P* > 0.05, ** = *P* < 0.05, *** = *P* < 0.001. ^a,b,c,^ Within a row, means without a common superscript letter differ (*P* ≤ 0.05)

**Table 3:** Effect of four different intranasal treatments on the serum IgG immune response (OD values ± s.d) values in Friesian calves

Treatments	Pre I	1 week PI	2 weeks PI	3 weeks PI	4 weeks PI	1 week PB	2 weeks PB	3 weeks PB	4 weeks PB	Half euthanised	5 days PE	12 days PE	2 weeks PE	3 weeks PE	T × S
	Day 0	Day 7	Day 14	Day 21	Day 28	Day 42	Day 49	Day 56	Day 63	Day 71	Day 76	Day 83	Day 85	Day 92	
Commercial vaccine	0.50^a^ ± 0.39	0.59^a^ ± 0.67	1.01^b^ ± 0.43	0.92^b^ ± 0.45	0.82^ab^ ± 0.45	0.76^a^ ± 0.45	0.94^b^ ± 0.39	1.04^b^ ± 0.42	1.39^c^ ± 0.46	1.47^c^ ± 0.57	1.28^c^ ± 0.48	1.25^bc^ ± 0.56	1.34^c^ ± 0.63	1.25^bc^ ± 0.66	**
Empty NPs	0.47^a^ ± 0.14	0.49^a^ ± 0.19	0.45^a^ ± 0.17	0.45^a^ ± 0.18	0.39^a^ ± 0.15	0.35^a^ ± 0.13	0.40^a^ ± 0.15	0.46^a^ ± 0.21	0.68^a^ ± 0.2	0.66^ab^ ± 0.2	0.72^b^ ± 0.27	0.54^ab^ ± 0.23	0.55^ab^ ± 0.25	0.51^ab^ ± 0.18	NS
BPI3V	0.46^a^ ± 0.16	0.69^a^ ± 0.26	0.72^a^ ± 0.31	0.65^a^ ± 0.23	0.64^a^ ± 0.24	0.69^a^ ± 0.22	0.81^a^ ± 0.26	1.10^b^ ± 0.52	1.10^b^ ± 0.46	1.22^bc^ ± 0.27	1.03^b^ ± 0.33	1.43^c^ ± 0.27	1.14^b^ ± 0.52	1.03^b^ ± 0.47	**
PLGA-BPI3V-NPs	0.43^a^ ± 0.12	0.78^b^ ± 0.44	0.75^b^ ± 0.45	0.69^b^ ± 0.4	0.62^b^ ± 0.33	0.72^b^ ± 0.47	0.77^b^ ± 0.47	0.96^bc^ ± 0.44	0.92^bc^ ± 0.38	1.06^c^ ± 0.42	1.14^c^ ± 0.36	1.11^c^ ± 0.43	1.04^c^ ± 0.46	1.13^c^ ± 0.54	**

*Pre I* Pre immunisation sampling, *PI* Post immunisation, *PB* Post booster, *PE* Post euthanasia, *T × S* treatment × sampling time interaction. Levels of significance: NS = *P* > 0.05, ** = *P* < 0.05, *** = *P* < 0.001. ^a,b,c,^ Within a row, means without a common superscript letter differ (*P* ≤ 0.05)

### Mucus IgA response

**Fig. 1:**
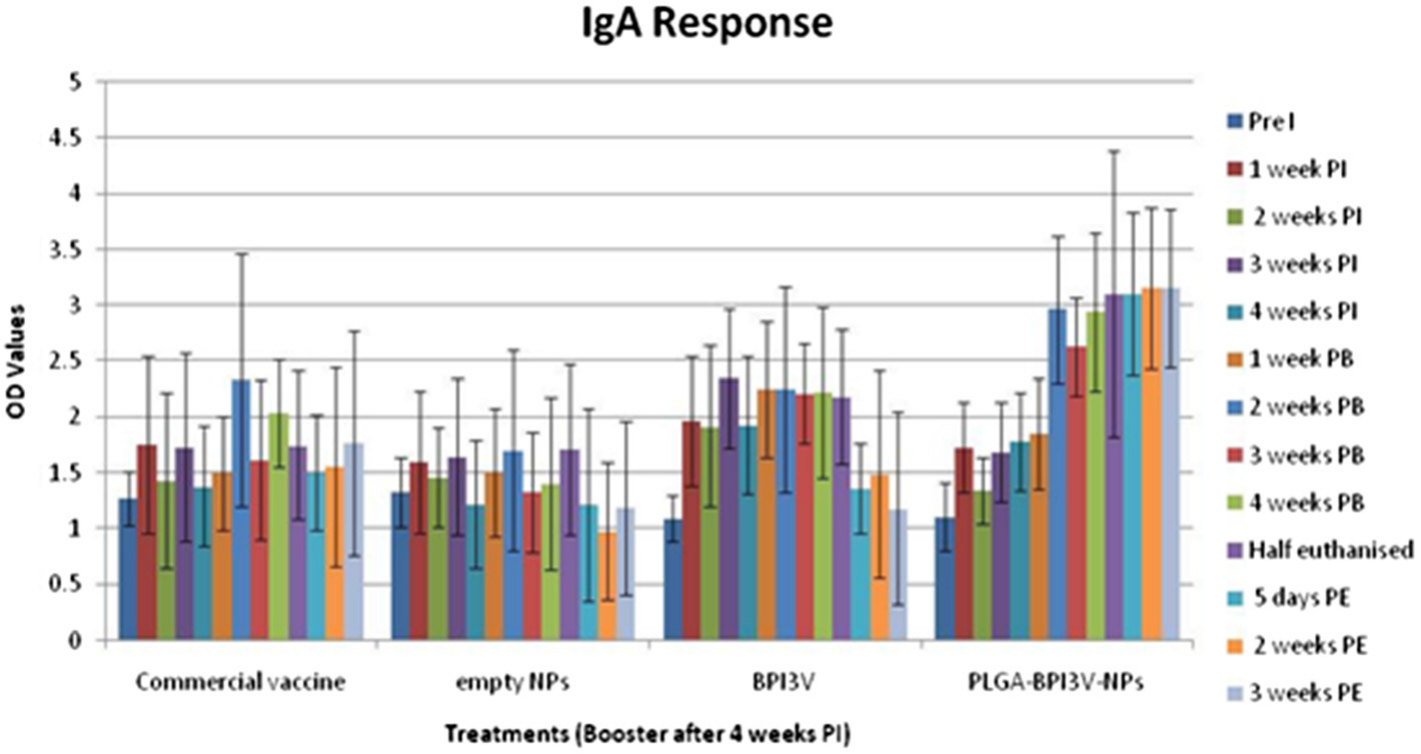
Mucosal IgA response to treatments; Figure showing mean (± s.d) mucosal IgA response (OD values) to four intranasal treatments: Commercial temperature-sensitive modified live virus intranasal vaccine; Empty - negative control (empty NPs); BPI3V -positive control (purified solubilised BPI3V proteins); and PLGA-BPI3V – NPs vaccine under test. Pre I = Pre immunisation sampling, PI = Post immunisation, PB = Post booster, PE = Post euthanasia (*n* = 3 left)

Mucus IgA levels in samples collected pre-immunisation were used as a covariate were not significantly different (*P* > 0.05) between the four treatment groups (Fig. 1, Table 2). There was a significant (*P* < 0.05) increase in virus-specific IgA in the nasal mucus of the group 4 calves given the PLGA-BP13V-NPs over the study period (Fig. 1, Table 2). This IgA response increased gradually and significantly (*P* < 0.05) following the ‘booster’ treatment, and continued to rise showing significant (*P* < 0.05) variation from the other three groups. These higher mucus IgA levels persisted until study completion (*P* < 0.05) at 92 days. Treatment with the commercial temperature-sensitive modified live virus vaccine (group 1) or the solubilised BPI3V (group 3), did not induce a statistically significant increase in mucus IgA over the study period to the end of the experiment. As expected the group 2 negative controls did not demonstrate any significant change in IgA levels over the study period.

### Serum-IgG-response

**Fig. 2:**
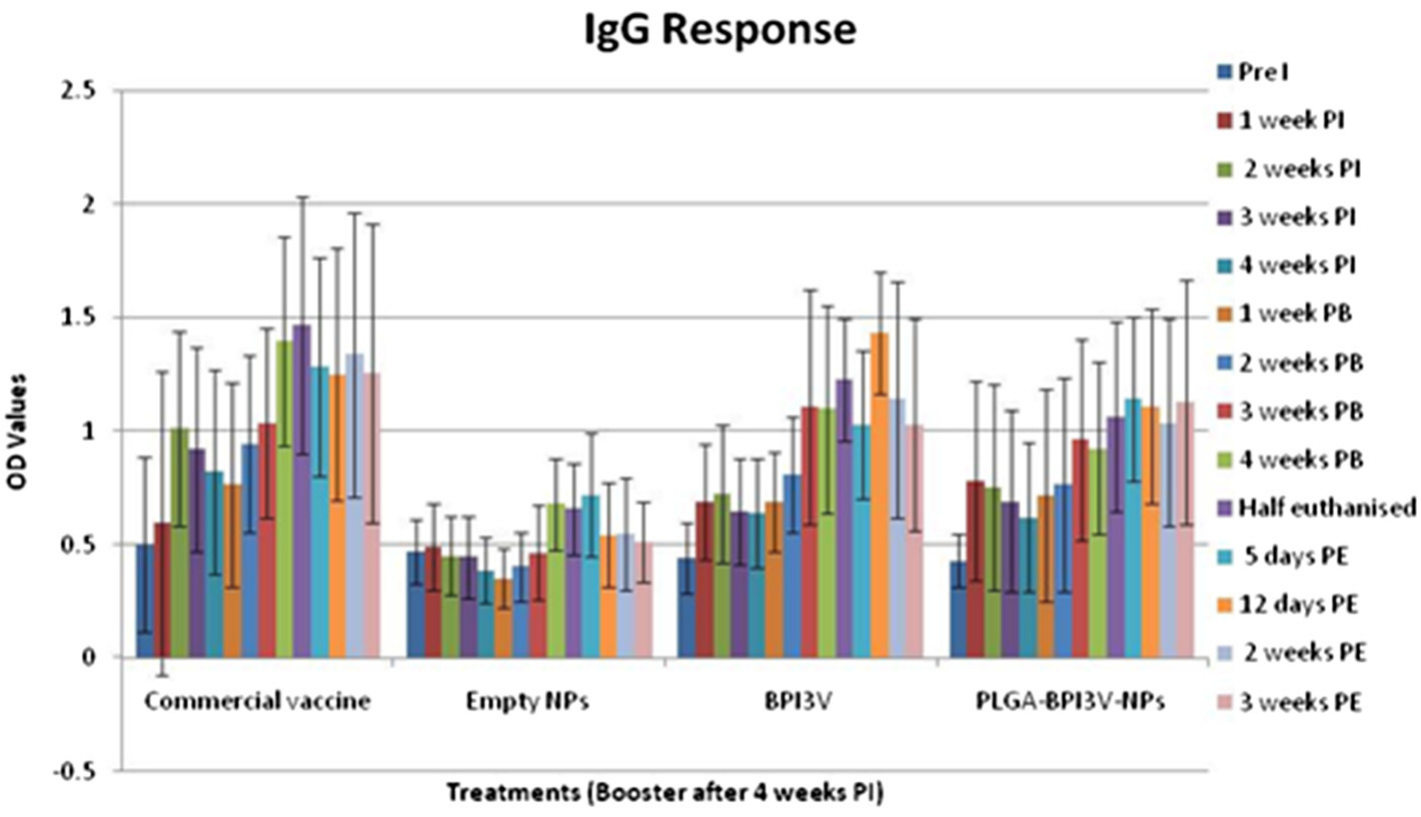
Serum IgG response to treatments; Figure showing mean (± s.d) serum IgG response (OD values) to four intranasal treatments: Commercial temperature-sensitive modified live virus intranasal vaccine; Empty - negative control (empty NPs); BPI3V -positive control (purified solubilised BPI3V proteins); and PLGA-BPI3V – NPs vaccine under test. Pre I = Pre immunisation sampling, PI = Post immunisation, PB = Post booster, PE = Post euthanasia (*n* = 3 left)

Serum IgG levels in samples collected pre-immunisation were similar (*P* > 0.05) between the four groups (Fig. 2). There was a significant increase in serum IgG concentration in groups (1, 3 and 4) relative to the negative controls group 2 (*P* < 0.05) (Fig. 2, Table 3) over the study period. The IgG response to PLGA-BPI3V-NPs (group 4) increased (*P* < 0.05) at 1 week post immunisation and increased gradually after the booster treatment and continued to rise showing a significant variation from the negative controls. Calves given the commercial vaccine (group 1) showed a significant rise (*P* ≤ 0.05) in IgG levels that continued to rise after the booster dose was administered. Calves in group 3 given solubilised BPI3V proteins demonstrated a significant (*P* < 0.05) rise in serum IgG levels from 3 weeks post booster onwards. There was a positive correlation (*P* < 0.001) between mucus IgA and serum IgG responses in all groups.

### Interferon (IFN)-γ response

There was no significant change (*P* > 0.05) in the release of IFN-γ from peripheral blood mononuclear cells (PBMC’s) in the four treatment groups over the duration of the study (data not shown).

## Discussion

The present study investigated the humoral and cellular immune responses of dairy calves (with pre-existing antibody responses to BPI3V) inoculated with a PLGA-BPI3V-NP vaccine and a commercial, temperature-sensitive modified live BPI3V vaccine. The calves given the PLGA-BPI3V-NPs had significantly greater mucus IgA responses, compared to the calves in the other three treatment groups following the booster treatment. This finding suggests an enhanced, more sustained mucosal-based immunological response to the candidate NP vaccine in the face of pre-existing systemic BPI3V-specific IgG and mucosal IgA. It is also established that surface charge or zeta potential is a critical parameter in controlling the development of stable and more functional nanoparticles. It is shown that negatively charged surface nanoparticles minimise nonspecific cellular uptake resulting in greater localised specific response [9]. Nanoparticles with negative zeta potential used in the current study could possibly be involved in greater localised BPI3V specific mucosal IgA response. The mucosal IgA response to PLGA-BPI3V-NPs observed in the current study is specific to heat inactivated purified whole BPI3V virus. However, in the future it would be worth investigating the mucosal IgA immune response towards more purified and concentrated immunodominant BPI3V antigens encapsulated in NPs along with assessing IgA response in combination with mucosal adjuvants.

The serum IgG responses in the PLGA-BPI3V-NPs treated calves were largely similar to those in the animals given the commercial vaccine, although they were lower between 4 and 5 weeks post-boosting. This could possibly be explained by the difference in the antigen release profiles from NPs as described by Kavanagh et al. [10] and the non-replicating format of the BPI3V NPs. The pre-existing BPI3V specific IgA and IgG antibodies in these animals were measured before the start of the study and the levels were largely similar in each of the treatment groups.

Given that the production of antibody at mucosal surfaces tends to be transient [7], the sustained presence of IgA in mucus on the URT surfaces of the calves given the PLGA-BPI3V-NPs suggests viral antigen presented in this form can result in a more sustained release of antigen over a longer period of time. Mucosal immunisation has become an important strategy in vaccine design as this approach stimulates both mucosal and systemic immune responses whereas parenteral immunisation typically results in only a systemic response [11]. Mucosal protection is particularly relevant in the context of respiratory pathogens where their major portal of entry is frequently the nasal mucosa [12, 13]. Mucosa-associated lymphoid tissue (MALT), an essential part of the mucosal immune system is the initial inductive site for nasal mucosal immunity. Antigens are sampled from mucosal surfaces and naïve B- and T-lymphocytes are stimulated. MALT structures (lymphoid follicles and T cell-dependent interfollicular areas) are the origin of lymphocyte migration to mucosal effector sites. Lymphatics transport immune cells and antigens to regional lymph nodes that are inductive sites of mucosa and augment the immune responses [14].

Sustained release of soluble antigen, as is likely to occur from NPs, has been shown to induce more robust immune responses than a larger single ‘spike’ or bolus of antigen given parenterally [15, 16]. In terms of potentially further enhancing the immunological impact of our candidate nanoparticle vaccine, muco-adhesive agents and immune potentiators/modulators such as chitosan, CpG motifs and MPLA could be used [17–21]. Muco-adhesive agents like chitosan has been shown to increase the penetration potential of the particles by increasing their adherence to mucosal membrane resulting in opening of tight junctions in the nasal mucosa.

No significant differences were detected in the concentrations of both nasal IgA and serum IgG specific BPI3V antibodies in any of the calves prior to study commencement and these Ig profiles are typical of commercially sourced colostrum-fed dairy calves [22, 23]. While these pre-existing antibodies may well have been maternally derived, the fact that the calves were not sampled until between 4 and 5 months of age means the possibility of prior exposure to BPI3V cannot be ruled out. However, there was no recent history of clinical respiratory disease on the farm where the calves were sourced.

The failure of the commercial vaccine to induce an anamnestic IgA and IgG antibody response instantly within a week of booster administration could possibly reflect the inhibitory effects of the circulating pre-existing antibodies as described previously [4, 24–27] which resulted in complete vaccine failure after a single dose and started to show some response after the second dose. Vangeel et al. [23] demonstrated that a single dose BPI3V vaccine did not provoke a significant rise in serum antibody levels in the presence of maternal antibodies and Greenberg et al. [26] reported that multiple doses of BPI3V vaccine were required to elicit protective immunity. Calves given the commercial live vaccine demonstrated significant systemic IgG responses 4–5 weeks after boosting. Such a finding has previously been reported in vaccine studies in cattle where efficacy of intranasal and intramuscular live attenuated respiratory vaccines was investigated [23, 26, 28, 29].

The smaller systemic IgG response in the PLGA-BPI3V-NPs treated animals relative to the commercial vaccine-treated calves was perhaps not surprising given the likely reduced quantity of inactive virus antigen in the NPs, relative to the ability of the live attenuated virus to replicate and therefore generate large quantities of antigen. In terms of pre-existing antibody interfering with vaccination, it is possible that pre-existing mucosal IgA could also negatively impact on conventional or NPs vaccines delivered onto mucocal surfaces. However as the NPs encapsulate the antigen it may be more likely that there would be less interference of existing antibodies on the development of an immune response towards vaccine NPs delivered by the aerosol route.

T-cells recognise antigenic peptides presented on MHC-encoded molecules and are fundamental to CMI. MHC-II-restricted T-cells (CD4) are known to have a primary role in the production of macrophage activating cytokines such as IFN-γ. IFN-γ enhances the ability of infected cells and phagocytes to inhibit the activity of infecting organisms. Interferon-γ can also be non-specifically produced by gamma-delta T and natural killer cells [30]. While some animals appeared to show IFN-γ responses at few occasions there were no consistent significant patterns correlating with vaccination. The antigen prepared for IFN-γ stimulation was a crude purified virus preparation and there could be other cellular antigens that may have caused some stimulation of cells to produce IFN-γ. Interestingly, enhanced mucosal IgA secretion has previously been linked to local IFN-γ production, where T cell products such as IFN-γ enhanced the translocation of large amounts of locally produced IgA through epithelial cells into mucosal secretions [31, 32].

## Conclusion

In conclusion, calves administered intranasal PLGA-BPI3V-NPs had significantly greater mucus IgA responses, compared to calves in the other three treatment groups. The increase in mucosal IgA response to NPs demonstrate the potential of vaccine delivery system that facilitates the slow release of antigen from the protective coating of PLGA NPs in the nasal mucosa. Of primary importance is that the antigen is protected from the deteriorative effects of mucosal enzymes and, being in the nano size range, has the potential to cross the mucosal barrier. Neutralizing respiratory viruses at the site of entry by eliciting local IgA response is crucial in young claves with a developing immune system which are subjected to an intensive milk management feeding system. Current study findings suggest an enhanced, sustained mucosal-based immunological response to the BPI3V nanoparticle vaccine in the face of pre-existing systemic IgG, which are encouraging and potentially useful characteristics of a candidate vaccine. This more sustained local mucosal immunity induced by the candidate PLGA-BPI3V-NPs vaccine has obvious potential if it translates into enhanced protective immunity in the face of virus outbreak.

## Methods

### Growth of BPI3V virus pools

A BPI3V isolate (2005/015033-Lung A), recovered from the pneumonic lung tissue of a 10 day old calf that was subject to post-mortem examination at the Agri-Food & Biosciences Institute (AFBI) in December 2005, was used to provide the necessary pools of virus. Canted, 75 cm^2^ neck flasks monolayered with foetal calf lung (FCL) cells of between 18 and 22 passages were obtained from the Cell Culture Laboratory at AFBI. Once a satisfactory monolayer of cells was visible upon microscopic examination, the cell maintenance medium was poured off and the flasks were infected with 2 ml of 1:300 diluted BPI3V working pool 1 (10^7.8^ tissue culture infectious dose 50 (TCID_50_)/ml) and incubated at 37 °C in 5 % CO_2_ for 1 h. Then, 18 ml of maintenance medium [Eagle’s BHK, 2 % heat inactivated foetal bovine serum, gentamicin (0.1 %) and glutamine (1 %)] was added to each flask and the inoculated monolayers incubated at 37 °C in 5 % CO_2_ for 7 days. The flasks were examined daily over 7 days for evidence of cytopathic effect (CPE).

### Harvesting of BPI3V

Following the 7 day incubation, extreme viral CPE (90 %) was evident and the flasks were transferred to a −80 °C freezer for 1 h. The flasks (16) were then thawed at 37 °C in a hot water bath and this freeze/thaw procedure was repeated three times. Cells were removed from the base of the flasks using a cell scrapper, pooled and placed in 400 ml glass bottles and stored at −80 °C prior to the virus purification step.

### BPI3V purification

An aliquot (320 ml) of the frozen virus pool (10^7.8^ TCID_50_/ml) was defrosted at 37 °C in a water bath. The thawed virus pool was dispensed into smaller bottles (80 ml in 100 ml bottles), chilled over ice, and sonicated (Sonics, Vibracell, USA) at full power for 1 min to disrupt any virus clumps before re-pooling into one glass bottle. The re-pooled virus suspension was heated in a water bath at 37 °C for 15 min and divided equally into polycarbonate centrifuge tubes (Corning, Mexico) each of which were spun down for 20 min at 1500 × g. The supernatant was decanted, leaving the cell pellet. The supernatant was then divided equally into polycarbonate tubes and balanced with 0.01 M PBS for ultracentrifugation (93,000 × g for 4 h at 4 °C). The virus pellets were re-suspended in 2 ml of PBS. The mixture was sonicated to create a suspension and the concentrated virus preparation was stored at −80 °C until required.

### Nanoparticle production

Purified BPI3V suspended in 2 ml of 0.01 M PBS was defrosted at room temperature. The virus suspension was heated at 70 °C for 10 min in a water bath for virus inactivation. Before encapsulating the viral proteins inside PLGA, gel electrophoresis was performed to assess the quality of the BPI3V proteins. Additionally, total protein mass in the virus suspension was calculated using the bicinchoninic acid (BCA) protein assay kit (Pierce, Thermo scientific, USA). For NP preparation, 1 ml of viral proteins (4 mg/ml) was emulsified with 300 mg of PLGA polymer dissolved in 8 ml of dichloromethane (DCM) (Sigma), 2 ml of acetone and 6 % Synperonic PE/F68 (FLUKA analytical, UK) using high-speed sonication at 55 % power for 2 min over ice. The emulsion was sonicated with 100 ml of 2.5 % emulsion stabiliser, polyvinyl alcohol (PVA) (Sigma) (2.5 g/100 ml distilled water) at 55 % power for 10 min over ice to form the final w/o/w solution [33, 34]. This final solution was left stirring overnight at room temperature in a fume hood followed by ultra-centrifugation (Thermo scientific Sorvall WX ultra series centrifuge, WX ultra 80) at 93,000 × g for 30 min at 4 °C. The pellets of NPs were washed three times by ultracentrifugation with distilled water (dH_2_O) and sonicated at full power (55 %) to obtain a uniform suspension before measuring the size and zeta potential of the NPs using Zetatrac (Microtrac, USA) equipment. The suspension was then freeze dried in a (Thermo ModulyoD) freeze drier under the conditions (pre-frozen sloped at −70 °C, placed in condenser chamber for 48 h keeping the temperature of condenser at −50 °C and pressure of chamber below 70 microbars). After freeze drying vials were sealed at 4 °C until required.

### Size and zeta potential measurement

The size and zeta potential (the charge residing on or near the surface of a suspended particle) of the NPs was measured using Zetatrac (Microtrac, USA). Zetatrac uses dynamic light scattering concept with high frequency electric field excitation and the interaction of random Brownian with driven electric field motion of particle suspensions to measure the size and zeta potential across the particles. A total of 3 ml of NP suspension in distilled H_2_O at the appropriate concentration and loading index was added to the measurement chamber of the Zetatrac instrument.

### Efficiency of encapsulation process

The content of encapsulated proteins in the NPs was determined using a bicinchoninic acid (BCA) protein assay kit (Pierce, Thermo scientific, USA). Approximately 10 mg of NPs were dissolved in 600 μl 0.1 M NaOH containing 5 % sodium dodecyl sulphate (BDH). The extraction was stopped after 4 h at 37 °C by adding 500 μl 0.1 M HCl. The solution was centrifuged to separate the proteins from the NPs. The supernatant was decanted and stored at −20 °C until analysed. The protein content was determined using a BCA protein assay kit [35–37] and a standard curve of known concentrations of bovine serum albumin (BSA) (25–2000 μg/ml) in an equal solution of 0.1 M NaOH containing 5 % SDS (sodium dodecyl sulfate) and 0.1 M HCl. The BCA working reagent was prepared by combining 50 parts of BCA stock solution and 1 part of 4 % cupric sulphate. Twenty five microlitres of each standard and supernatant from the NPs extraction procedure was added in duplicate to a 96-well plate (Sterilin, UK). Two hundred microlitres of working BCA solution was then added into each well. The microtitre plate was covered with a plate sealer and incubated for 30 min at 37 °C with gentle shaking. After 30 min of incubation, the plate was cooled to room temperature and the absorbance of water-soluble purple product was measured at 562 nm using a microplate reader (Tecan Sunrise, Switzerland).

### Antibody detection

#### IgA ELISA

Bovine parainfluenza virus type 3-specific IgA in nasal washings was detected by ELISA. Ninety-six-well ELISA plates (Immulon 1B, Dynex Technologies) were coated overnight with purified BPI3V as described above by adding 100 μl per well of a solution of 1 μl/4 ml of purified BPI3V in 0.05 M sodium carbonate–bicarbonate buffer (pH 9.6) at 4 °C. After 24 h, the plates were washed three times with wash solution containing Tris (6 g/l), NaCl (8.19 g/l) and 0.05 % Tween 20. A total of 200 μl of wash solution containing Tris, NaCl and Tween was then added to the wells and incubated for 30 min at 37 °C. The plates were washed three times with wash solution. The nasal mucus samples were diluted 1:4 in conjugate diluent (Bethyl Laboratories, Inc., USA) of same composition as of wash solution containing 0.05 % Tween 20 and 100 μl of each sample was added in duplicate to the wells of the coated plates. The plates were incubated for 1 h at 37 °C. The plates were then washed five times with wash solution. One hundred microlitres of rabbit anti-bovine IgA horseradish peroxidase (HRP) conjugate (Bethyl Laboratories, Inc. USA), diluted 1:10,000 in conjugate diluent containing 4 % horse serum (to prevent non-specific binding) was added to each well and the plates were again incubated for 1 h at 37 °C followed by washing as previously described. One hundred microlitres of the enzyme substrate (TMB) (Bethyl Laboratories, Inc. USA) were added to each well, followed by a 15 min incubation at room temperature in the dark with colour development terminated by adding 100 μl of 0.18 M hydrosulphuric acid (H_2_SO_4_). Optical density (OD) values were read at 450 nm using an ELISA reader (Tecan Sunrise, Switzerland).

### IgG ELISA

A BPI3V antibody ELISA kit (Svanovir, Scandinavia) was used to analyze serum IgG. Briefly, serum samples were diluted 1/25 in PBS-Tween buffer and applied to the wells for 1 h at 37 °C, before washing three times with PBS-Tween and applying anti-bovine IgG conjugate (provided in the kit) for 1 h. The substrate and ‘stop’ solution used were also supplied with the ELISA kit. Absorbance was read at 450 nm using an ELISA reader (Tecan Sunrise, Switzerland).

### Stimulated lymphocyte production of interferon-γ (IFN-γ)

The stimulated lymphocyte production of IFN-γ was determined from harvested supernatant following a whole-blood culture [38]. Heparinised blood samples (10 ml) were collected from animals and cultured in sterile 96-well flat culture plates (200 μl whole blood/well). For non-stimulated controls, 25 μl of PBS was added to wells and for antigen-specific stimulation, a total of 25 μl of BPI3V antigen in PBS was added (final antigen concentration of 4 μg/ml). As a positive control, pokeweed mitogen (PWM) was added (final concentration 4 μg/ml). Control or stimulated wells were established in duplicate and the cell culture was incubated for 24 h at 37 °C in an atmosphere of 5 % CO_2_ in air. Aseptic techniques were practiced during this procedure. The culture plates were then centrifuged at 1,700 × g, at 8 °C for 20 min; the supernatant was harvested and stored frozen at −20 °C until assayed. The supernatant was assayed using the BOVIGAM® gamma interferon ELISA for cattle according to the manufacturer’s guidelines (Prionics, Switzerland). The IFN-γ response was determined by comparing the net OD for each sample.

### Experimental animals

Twenty four weaned Friesian bull calves were commercially sourced at day 0 (mean age 141 [s.d, 8.8 days] at day 0, mean weight 121.8 [s.d, 21.4] kg). Calves were identified by individual ear tag number and housed in open-fronted straw-bedded sheds. The calves were weighed seven days before the immunisation procedure. Water was provided *ad libitum*. Calves were fed high quality haylage *ad libitum* and 1.5 kg per/head/ day of 16 % calf rearing ration. Calves were immunised 83 [s.d, 7.15] days after weaning (day 0).

### Experimental treatments

All calves were blood and nasal mucus sampled on day 0, to determine the presence of BPI3V specific pre-existing IgG and IgA in serum and mucus, respectively. There was no history of respiratory outbreaks on the farm from where the calves came and the vaccines had no history of vaccination for BRD. The calves were clinically examined and declared fit for the study on day 0 by the Named Veterinary Surgeon and were randomly assigned based on live-weight to one of the following four treatment groups with six calves in each treatment group.

1. Commercial intranasal vaccine group – given temperature-sensitive, modified live virus vaccine (Rispoval RS + PI3 intranasal, Pfizer) was given according to manufacture instructions i.e. 1 ml in each nostril and boosted at day 35.
2. Negative control group – given empty PLGA-NPs.
3. Positive control group – given purified BPI3V proteins (1.3 mg of BPI3V protein/2 ml of PBS
4. PLGA-BPI3V-NPs (1.3 mg/2 ml of PBS).

Fifteen minutes prior to immunisation of the calves on day 0, the freeze dried NPs were suspended in 0.01 M PBS and mixed thoroughly for 1 min by vortexing. Briefly, 1 ml of each suspension was inoculated into each nostril of each calf using an intranasal spray applicator (Pfizer). The total volume administered was 2 ml/calf. Calves were restrained in a crush with the head held in an upright position, nostrils facing upwards for 30 s after each inoculation. Blood was collected from the jugular vein and nasal mucus from the nostrils weekly over the next 4 weeks (Table 4). Mucus was collected using nasal sponges inserted into each nostril for 1 min and then squeezed out using a sterile syringe into a 5 ml sterile collection tube. Nasal mucus was centrifuged to remove particulate debris. Nasal mucus and serum were screened for mucosal IgA and serum IgG concentrations, respectively using ELISA as mentioned in section 5.7.1 and 5.7.2.

**Table 4:** Timeline showing timings of blood and nasal sample collection from calves

Intranasal treatments (6 animals/group)			
Commercial Vaccine	Empty NPs	BPI3V proteins	PLGA-BPI3V-NPs
Blood collection	Nasal mucus collection		
Day 0 (Immunisation)	Day 0 (Immunisation)		
Day 7 (1 week PI)	Day 7 (1 week PI)		
Day 14 (2 weeks PI)	Day 14 (2 weeks PI)		
Day 21 (3 weeks PI)	Day 21 (3 weeks PI)		
Day 28 (4 weeks PI)	Day 28 (4 weeks PI)		
Day 35 (Booster inoculation)	Day 35 (Booster inoculation)		
Day 42 (1 week PB)	Day 42 (1 week PB)		
Day 49 (2 weeks PB)	Day 49 (2 weeks PB)		
Day 56 (3 weeks PB)	Day 56 (3 weeks PB)		
Day 63 (4 weeks PB)	Day 63 (4 weeks PB)		
Day 71 (n = 3, euthanised)	Day 71 (n = 3, euthanised)		
Day 76 (5 days PE)	Day 76 (5 days PE)		
Day 83 (12 days PE)			
Day 85 (2 weeks PE)	Day 85 (2 weeks PE)		
Day 92 (3 weeks PE)	Day 92 (3 weeks PE)		

*PI* Post immunisation, *PB* Post booster, *PE* Post euthanasia

Whole blood samples were collected and stimulated as described above for the production of IFN-γ. Calves were administered a booster immunisation in the same manner as the first immunisation dose on day 35 including commercial vaccine and the same parameters were measured weekly for a further 5 weeks (Table 4). Half of the calves from each group were necropsied on day 76. Blood and nasal mucus from remaining calves was collected for the following 3 weeks (Table 4) to determine the levels of serum IgG, mucus IgA and IFN-γ production.

### Statistical analysis

Genstat statistical software was used to analyse the data. Repeated measure ANOVA was used to analyse differences among treatments at individual bleeds and over the study period. Pre-immunisation blood and nasal mucus were used as covariates in the analysis, and P < 0.05 was considered significant. Fisher’s protected least significant difference test was used to determine differences among the same and different treatments at different sampling times. Correlation analysis was performed to determine the relationship between IgG, IgA, and IFN-γ concentrations.

### Animal experimentation ethics

All animal studies were carried out in accordance with the UK Animals (Scientific Procedures) Act 1986. Approval was granted by the local AFBI Ethical Review Committee and a formal project license was approved by the regulatory authorities.

## Abbreviations

ANOVA: Analysis of variance
BCA: Bicinchoninic acid
BHK: Baby hamster kidney
BPI3V: Bovine Parainfluenze-3 Virus
BRD: Bovine Respiratory Disease
BRSV: Bovine respiratory syncytial virus
BSA: Bovine serum albumin
BVDV: Bovine viral diarrhoea virus
°C: Degrees centigrade
CD4: T helper cells
CMI: Cell mediated immunity
CO_2_: Carbon dioxide
CPE: Cytopathic effect
CpG-ODN: CpG oligodeoxynucleotides
DCM: Dichloromethane
dH_2_O: Distilled water
DNA: Deoxyribonucleic acid
ELISA: Enzyme-linked immunosorbent assay
FCL: Fetal calf lung cells
×g: Gravitiational acceleration
H_2_SO_4_: Hydrosulphuric acid
HCL: Hydrochloric acid
IFN-γ: Interferon gamma
Ig: Immunoglobulin
MALT: Mucosa associated lymphoid tissue
MHC: Major histocompatibility complex
MPLA: Monophosphoryl lipid A
NaCl: Sodium chloride
NaOH: Sodium hydroxide
NPs: Nanoparticles
NS: Not statistically significant
OD: Optical density
PBMCs: Peripheral blood mononuclear cells
PBS: Phosphate buffer saline
PEG: Poly (ethylene glycol)
PLGA: Poly (lactic co glycolic acid)
PLGA-BPI3V-NPs: Poly (lactic co glycolic acid) nanoparticles encapsulating bovine parainfluenza-3 Virus
PLGA-NPs: Poly (lactic co glycolic acid) nanoparticles
PVA: Polyvinyl alcohol
PWM: Pokeweed mitogen
Pre: I pre-immunisation
PI: Post immunisation
RT: Room temperature
s.d.: Standard deviation
TCID: Tissue culture infectious dose
Th: Helper T cells
T: Treatment
T × S: Treatment × sampling time interaction
URT: Upper respiratory tract
w/o/w: Water in oil in water

## References

[1] Adair BM (2009). Nanoparticle vaccines against respiratory viruses. Wiley Interdiscip Rev Nanomed Nanobiotechnol.

[2] Crowe JE (2001). Influence of maternal antibodies on neonatal immunization against respiratory viruses. Clin Infect Dis.

[3] Menanteau-Horta A, Ames T, Johnson D, Meiske J (1985). Effect of maternal antibody upon vaccination with infectious bovine rhinotracheitis and bovine virus diarrhea vaccines. Can J Comp Med.

[4] Kimman TG, Westenbrink F, Schreuder BEC, Straver PJ (1987). Local and systemic antibody-response to bovine respiratory syncytial virus-infection and reinfection in calves with and without maternal antibodies. J Clin Microbiol.

[5] Morein B, Abusugra I, Blomqvist G (2002). Immunity in neonates. Vet Immunol Immunopathol.

[6] Morein B, Blomqvist G, Hu K (2007). Immune responsiveness in the neonatal period. J Comp Pathol.

[7] Shewen PE, Carrasco-Medina L, McBey BA, Hodgins DC (2009). Challenges in mucosal vaccination of cattle. Vet Immunol Immunopathol.

[8] Mansoor F, Earley B, Cassidy JP, Markey B, Foster C, Doherty S (2014). Intranasal delivery of nanoparticles encapsulating BPI3V proteins induces an early humoral immune response in mice. Res Vet Sci.

[9] Alexis F, Pridgen E, Molnar LK, Farokhzad OC (2008). Factors affecting the clearance and biodistribution of polymeric nanoparticles. Mol Pharm.

[10] Kavanagh OV, Earley B, Murray M, Foster CJ, Adair BM (2003). Antigen-specific IgA and IgG responses in calves inoculated intranasally with ovalbumin encapsulated in poly(DL-lactide-co-glycolide) microspheres. Vaccine.

[11] Chen HM (2000). Recent advances in mucosal vaccine development. J Control Release.

[12] Zinkernagel RM, Ehl S, Aichele P, Oehen S, Kündig T, Hengartner H (1997). Antigen localisation regulates immune responses in a dose‐and time‐dependent fashion: a geographical view of immune reactivity. Immunol Rev.

[13] Zinkernagel RM. Localization dose and time of antigens determine immune reactivity. In: 2000: Elsevier; 2000: 163–71. 10.1006/smim.2000.0253 10910735

[14] Liebler-Tenorio EM, Pabst R (2006). MALT structure and function in farm animals. Vet Res.

[15] Higaki M, Azechi Y, Takase T, Igarashi R, Nagahara S, Sano A (2001). Collagen minipellet as a controlled release delivery system for tetanus and diphtheria toxoid. Vaccine.

[16] Lofthouse S (2002). Immunological aspects of controlled antigen delivery. Adv Drug Deliv Rev.

[17] Garinot M, Fievez V, Pourcelle V, Stoffelbach F, des Rieux A, Plapied L (2007). PEGylated PLGA-based nanoparticles targeting M cells for oral vaccination. J Control Release.

[18] Buonaguro FM, Tornesello ML, Buonaguro L (2011). New adjuvants in evolving vaccine strategies. Expert Opin Biol Ther.

[19] Kwon YJ, Standley SM, Goh SL, Frechet JMJ (2005). Enhanced antigen presentation and immunostimulation of dendritic cells using acid-degradable cationic nanoparticles. J Control Release.

[20] Vila A, Sanchez A, Perez C, Alonso MJ (2002). PLA-PEG nanospheres: New carriers for transmucosal delivery of proteins and plasmid DNA. Polymer Adv Tech.

[21] Tahara K, Sakai T, Yamamoto H, Takeuchi H, Hirashima N, Kawashima Y (2009). Improved cellular uptake of chitosan-modi fled PLGA nanospheres by A549 cells. Int J Pharm.

[22] Bryson DG, Adair BM, McNulty MS, McAliskey M, Bradford HEL, Allan GM (1999). Studies on the efficacy of intranasal vaccination for the prevention of experimentally induced parainfluenza type 3 virus pneumonia in calves. Vet Rec.

[23] Vangeel I, Ioannou F, Riegler L, Salt JS, Harmeyer SS (2009). Efficacy of an intranasal modified live bovine respiratory syncytial virus and temperature-sensitive parainfluenza type 3 virus vaccine in 3-week-old calves experimentally challenged with PI3V. Vet J.

[24] Siegrist CA, Cordova M, Brandt C, Barrios C, Berney M, Tougne C (1998). Determinants of infant responses to vaccines in presence of maternal antibodies. Vaccine.

[25] Lee MS, Greenberg DP, Yeh SH, Yogev R, Reisinger KS, Ward JI (2001). Antibody responses to bovine parainfluenza virus type 3 (PIV3) vaccination and human PIV3 infection in young infants. J Infect Dis.

[26] Greenberg DP, Walker RE, Lee MS, Reisinger KS, Ward JI, Yogev R (2005). A bovine parainfluenza virus type 3 vaccine is safe and immunogenic in early infancy. J Infect Dis.

[27] Bradshaw BJF, Edwards S (1996). Antibody isotype responses to experimental infection with bovine herpesvirus 1 in calves with colostrally derived antibody. Vet Microbiol.

[28] Peters AR, Thevasagayam SJ, Wiseman A, Salt JS (2004). Duration of immunity of a quadrivalent vaccine against respiratory diseases caused by BHV-1, PI3V, BVDV, and BRSV in experimentally infected calves. Prev Vet Med.

[29] Salt JS, Thevasagayam SJ, Wiseman A, Peters AR (2007). Efficacy of a quadrivalent vaccine against respiratory diseases caused by BHV-1, PI(3)V, BVDV and BRSV in experimentally infected calves. Vet J.

[30] Pollock J, Welsh M (2002). The WC1+ [gamma][delta] T-cell population in cattle: a possible role in resistance to intracellular infection. Vet Immunol Immunopathol.

[31] Phillips J, Everson M, Moldoveanu Z, Lue C, Mestecky J (1990). Synergistic effect of IL-4 and IFN-gamma on the expression of polymeric Ig receptor (secretory component) and IgA binding by human epithelial cells. J Immunol.

[32] Pilette C, Ouadrhiri Y, Godding V, Vaerman JP, Sibille Y (2001). Lung mucosal immunity: immunoglobulin-A revisited. Eur Respir J.

[33] Song CX, Labhasetwar V, Murphy H, Qu X, Humphrey WR, Shebuski RJ (1997). Formulation and characterization of biodegradable nanoparticles for intravascular local drug delivery. J Control Release.

[34] Hans ML, Lowman AM (2002). Biodegradable nanoparticles for drug delivery and targeting. Curr Opin Solid State Mater Sci.

[35] Smith PK, Krohn RI, Hermanson GT, Mallia AK, Gartner FH, Provenzano MD (1985). Measurement of protein using bicinchoninic acid. Anal Biochem.

[36] Wiechelman KJ, Braun RD, Fitzpatrick JD (1988). Investigation of the bicinchoninic acid protein assay - identification of the groups responsible for color formation. Anal Biochem.

[37] Brown RE, Jarvis KL, Hyland KJ (1989). Protein measurement using bicinchoninic acid - elimination of interfering substances. Anal Biochem.

[38] Wood PR, Corner LA, Plackett P (1990). Development of a simple, rapid invitro cellular-assay for bovine tuberculosis based on the production of gamma-interferon. Res Vet Sci.

